# Wire Arc Additive Manufacturing of Al-Mg Alloy with the Addition of Scandium and Zirconium

**DOI:** 10.3390/ma14133665

**Published:** 2021-06-30

**Authors:** Taisiya Ponomareva, Mikhail Ponomarev, Arseniy Kisarev, Maxim Ivanov

**Affiliations:** S7 R&D Center, 5, Vostochnaya Street, 142712 Gorki Leninskiye, Russia; m.a.ponomarev@s7.ru (M.P.); a.kisarev@s7.ru (A.K.); m.b.ivanov@s7.ru (M.I.)

**Keywords:** wire arc additive manufacturing (WAAM), additive manufacturing, cold metal transfer (CMT), aluminum scandium alloys (Al-Sc), scandium, hardness, mechanical properties, cooling rate

## Abstract

The proposed paper considers the opportunity of expanding the application area of wire arc additive manufacturing (WAAM) method by means of increasing the strength properties of deposited material, due to the implementation of aluminum wire with the addition of scandium and zirconium. For the experimental research, the welding wire 1575 of the Al-Mg-Sc-Zr system containing 0.23% Sc and 0.19% Zr was selected. The optimal welding parameters, ensuring the defect-free formation of deposited material with low heat input, were used. Porosity level was estimated. The thermal state was estimated by finite element simulation. Simulated thermal state was verified by comparison with thermocouples data. Post-heat treatment parameters that lead to maximum strength with good plasticity were determined. The maximum yield strength (YS) of 268 MPa and ultimate strength (UTS) of 403 MPa were obtained, while the plasticity was determined at least 16.0% in all WAAM specimens.

## 1. Introduction

The wire arc additive manufacturing process (WAAM) is a technology to produce large-sized metal products, including in the aerospace industry [1,2,3]. WAAM allows the production of complex structures with a high material utilization rate. The WAAM product has a cast structure, which is characterized by low mechanical properties. Increasing the mechanical properties of WAAM products will expand their potential application for high-load parts. The actual tasks for the study of WAAM are the choice of filler wire, arc modes, and methods of strengthening in order to obtain products with high mechanical properties. In [4], two strengthening methods of 2319 alloy deposits were investigated—inter-layer cold working and post-deposition heat treatment. The UTS and YS of the inter-layer rolled 2319 alloy achieve 314 MPa and 244 MPa, respectively, whereas, by heat treatment, these values exceed 450 MPa and 305 MPa. In [5], the UTS of AA2050 alloy deposits after post-deposited heat treatment exceeds 400 MPa. In [6], after heat treatment, the YS of Al-Mg-0.3Sc achieves 279MPa. In [7], the maximum UTS of 333 MPa of Al-6Mg alloy deposits was achieved due to the use of the VP-CMT arc mode. In [8], the YS of 5183 + 0.41 Sc alloy after heat treatment achieves 259 MPa.

The base structural materials in the aerospace industry are non-heat-treatable alloys of the Al-Mg system, whose high levels of strength properties can be obtained by mechanical hardening (cold rolling, stamping, etc.). The mechanical properties of WAAM deposits are close to the properties of these alloys’ welded joints [7]. The addition of scandium and zirconium to the Al-Mg system is a well-known method of strengthening these alloys [9,10,11,12,13,14]. Scandium is one of the best modifiers of the cast grain structure; the addition of zirconium enhances and stabilizes the effect of scandium. Strengthening occurs as a result of artificial aging by the formation of nanosized Al_3_Sc precipitates at the decomposition of the supersaturated solid solution at a sufficiently high (>100 °C/s) crystallization rates [15,16]. The solid solution of scandium in aluminum is unstable, and decomposes at the temperature of 250 °C [15,17]. The best level of mechanical properties is achieved due to the formation of Al_3_Sc precipitates with sizes of 2–6 nm in diameter at temperatures between 250 °C and 400 °C [15]. Higher heating temperatures decrease the strength of the alloy due to a partial coherence loss of the Al_3_Sc phase particles [18].

This paper presents the opportunity of expanding the application area of the WAAM method due to the application of Al-Mg-Sc-Zr system welding wire (Sv1575), which can be classified as 5XXX group of alloys (Table 1). It is very important to achieve the increase in mechanical properties without additional cost. For this reason, the welding wire with mutual alloying of Sc and Zr was selected. The application of Sc is limited due to its high cost, while the addition of Zr allows using the Sc with its lower concentrations. Additionally, this paper considers the influence of heat treatment on the strengthening degree of the WAAM deposits to estimate optimal parameters. Although the strengthening of Al-Mg alloys due to scandium and zirconium is well studied, the possibility of obtaining high mechanical properties on WAAM deposits is very rarely found in the literature [8]. To predict the possibility of obtaining a supersaturated solid solution after deposition, that is prone to further aging, the cooling rate is to be estimated.

## 2. Materials and Methods

**Figure 1:**
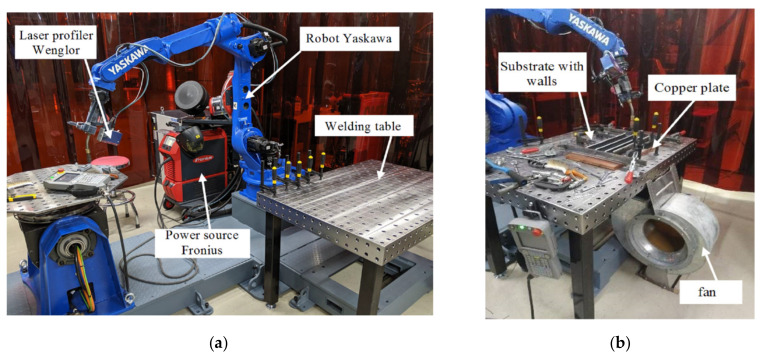
(**a**) Experimental setup for WAAM; (**b**) substrate with four deposited walls.

In this study, the deposition of thin walls using CMT method (Cold Metal Transfer) was carried out in order to determine heat treatment influence on the degree of strengthening of the deposited metal. The additive manufacturing system included a Fronius TPS 500i CMT arc welding power source (Fronius Gmbh, Wels, Austria) on process line #3727 and an Yaskawa MA2010 welding robot (Figure 1, Yaskawa Nordic Ab, Jönköping, Sweden).

The walls were deposited on Al-6Mg substrate (Table 1) with dimensions 500 mm × 300 mm × 6 mm, and mounted on a 600 mm × 300 mm × 20 mm copper plate. A tight contact between the substrate and the copper plate was not ensured. Additional cooling was continuously applied during experiment by the fan located on the bottom side of the table. The welding parameters with the low heat input were used as a basis [19,20]. The walls had a height of at least 40 mm and a thickness of more than 5 mm, to obtain specimens for tensile testing.

**Figure 2:**
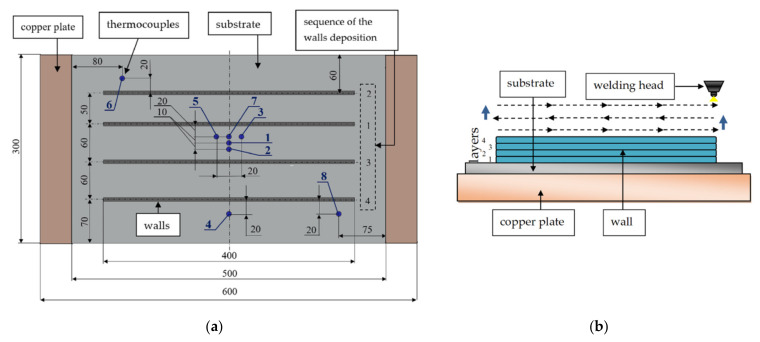
(**a**) Thermocouples location and the sequence for walls manufacturing (all dimensions are in millimeters); (**b**) deposition direction.

The sequence of walls deposition is shown in Figure 2a. Each wall was completely deposited; the next one was deposited after a pause to cool the substrate to 20 °C. Each layer was carried out for the entire length of the wall (400 mm). The deposition was carried out with pauses between the layers; the deposition direction was changed to the opposite from layer to layer (Figure 2b). A pause was maintained between the beads to cool the substrate to a temperature of less than 100 °C. There were 8 walls deposited on two substrates overall. The welding parameters and the pause time between the layers are presented in Table 2.

To estimate the cooling rate during deposition, a finite element simulation was carried out using Ansys software (ANSYS Mechanical Enterprise, ANSYS Inc, Canonsburg, PA, USA). The simulation was performed for the first weld bead, next to which thermocouples were located. Other weld beads were eliminated from the simulation as their location from thermocouples was too far from the heat source.

**Figure 3:**
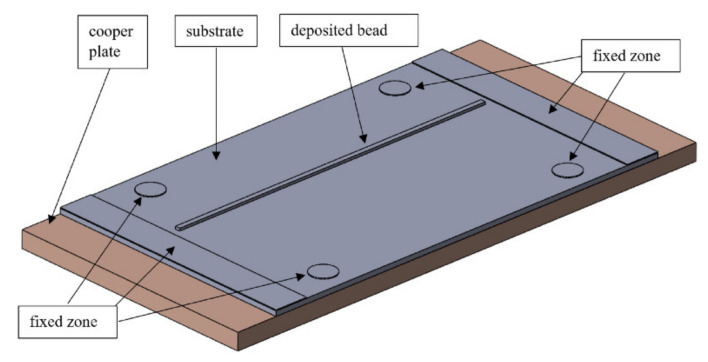
Geometrical model.

The geometrical model is shown in Figure 3 and consists of an M1 grade cooper plate, a substrate from Al-6Mg alloy, and a deposited bead from 1575 alloy. The simulation model was sectioned on separated volumes to build the uniform mesh and set up boundary conditions. Mesh consisted of 20 nodes of quadratic elements (SOLID186); the size of the elements near weld bead was 1 mm, while the elements in the far area had an average size of 5 mm.

The total heat power of the heat source during deposition was equal to arc welding power, as shown in the following Equation (1):

(1) Q=η·I·U

where η=0.95—arc efficiency coefficient; U—arc voltage, V; and I—arc current, A. The values of these variables were obtained from Table 2 for the first bead.

Thermophysical properties of materials were set from the Ansys database. The thermal condition of the deposition was estimated by the cooling rate of the weld pool boundary. The estimation was carried out by comparing the calculated and experimental data. The data obtained from the thermocouples were used to adjust the power density distribution of the heat source. The power of the heat source was described by a normal distribution model (2) with different distributions along and across the bead in -X and -Y directions [21]:

(2) $$q\left(x,y\right)=\frac{3\cdot Q}{\pi \cdot R_{x}\cdot R_{y}}\cdot exp\left(-\frac{3}{R_{x}^{2}}\cdot x^{2}-\frac{3}{R_{y}^{2}}\cdot y^{2}\right)$$

where $q\left(x,y\right)$—heat power density distribution, $W/{\mathrm{mm}}^{2}$; Q=η·I·U—welding arc power, W; $R_{x},\;R_{y}$—heat source distribution boundaries along the axes, mm; and *x*, *y*—distance from the heat source to point, mm.

**Figure 4:**
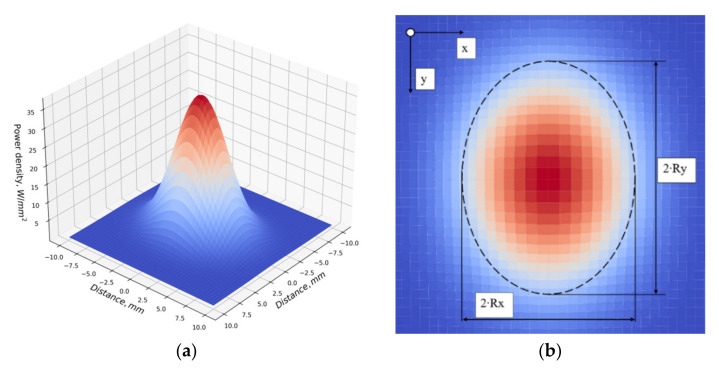
Power density distribution of the heat source: (**a**) 3D power density distributions of the heat source; (**b**) source distribution zone parameters.

The parameters of the heat source for Formula (2) are presented in Table 3. A power density distribution visualization of the heat source is shown in Figure 4.

**Figure 5:**
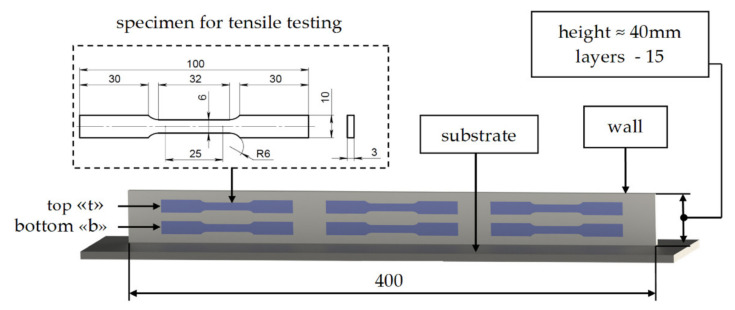
Specimens location in wall and one’s dimensions (all dimensions are in millimeters).

The deposited metal of the walls was subjected to tensile testing and hardness measurements. Specimens for tensile testing were prepared according to ASTM E8/E8M-16ae1 [22], and were sectioned from thin-walls, as shown in Figure 5. Three specimens in length and two in height were made from each wall, with the corresponding marking: “t” and “b” to indicate the top and the bottom relative to the substrate.

Specimens were aged in the temperature range of 275–350 °C with a step of 25 °C for 1–12 h in a SNOL 1100/30 muffle furnace (Umega Group, AB SnolTherm, Narkūnai (Utena), Lithuania). The specimens were placed in the furnace on an aluminum table with an already steady-stated temperature. The temperature in the furnace was controlled by three thermocouples (the top and the center of the chamber, and the aluminum table). The temperature was in range from −5 to +10 °C from the set level during the aging process.

Tensile testing was performed on a universal tensile testing machine, MTS Criterion 43 (MTS Systems Corporation, Eden Prairie, MN, USA), with a maximum load capacity of 50 kN. The Vickers hardness was measured using a KB 50SR microhardness tester (KB Prüftechnik GmbH, Hochdorf-Assenheim, Germany). Ten measurements with a load of 1 kg were carried out on each specimen.

Metallographic examinations were carried out on specimens with and without heat treatment after deposition. The thin sections were etched by Keller’s reagent (HF 20%, HCl 30%, H_2_O 50%) to reveal the structure. The examinations were carried out using a Leica DMi8 optical microscope (Leica Microsystems GmbH, Wetzlar, Germany).

The porosity was evaluated using the ImageJ image analysis program (1.52a, Rasband, W.S., National Institutes of Health, Bethesda, MD, USA). The total number and area of pores in the cross-sectional of the specimens were determined. The minimum threshold value of the pore area was 0.0001 mm^2^ (diameter 11 μm).

## 3. Results and Discussions

There were 42 specimens, obtained from seven walls. One wall with six specimens was rejected as it was damaged during the milling process due to operator error. The deposition of the layers of each wall was carried out in the same conditions that are confirmed by the results of temperature recording using thermocouples. It was determined that the temperature was reduced to less than 70 °C before the start of the next layer by deposition with pauses.

**Figure 6:**
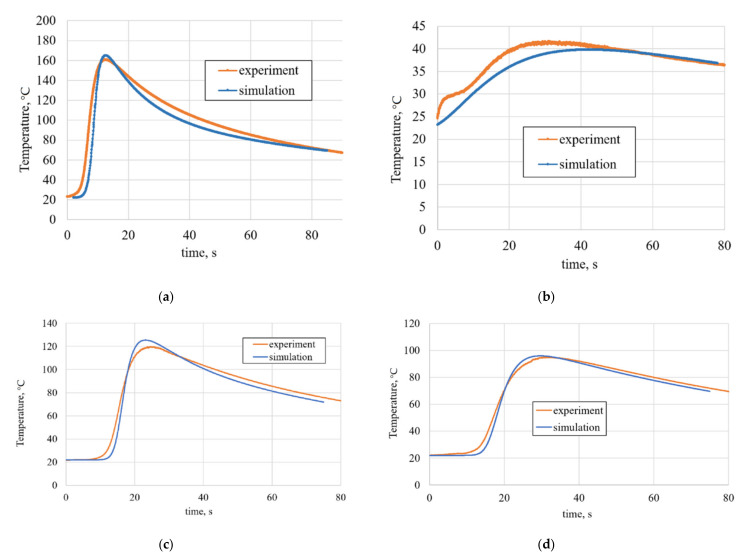

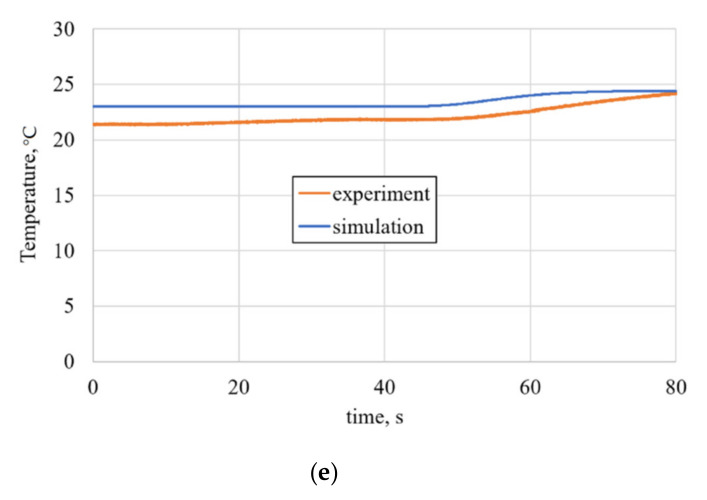
Thermal cycles for thermocouples: (**a**) № 7; (**b**) № 6; (**c**) № 1; (**d**) № 2; and (**e**) № 8.

Calculation of temperature fields from the action of the heat source, with the parameters from Table 3, demonstrated a good agreement with experimental data. The comparison of experimental and simulations data for thermocouples № 6, № 7, № 1, № 2, and № 8 is presented in Figure 6.

The first wave of the experimental result in Figure 6b can be explained by two inaccuracies during FEM analysis: (1) not an accurate enough heat flow reflection coefficient in case of top plate heat reflection. In this case, we observe heat accumulation from plate top in a larger amount than we expected. (2) not an accurate enough description of contact between aluminum substrate and copper plate. As the substrate was not in full area tight contact with the copper plate (only in fixture places), this may lead to FEM analysis deviation, especially in distant from heat source elements.

**Figure 7:**
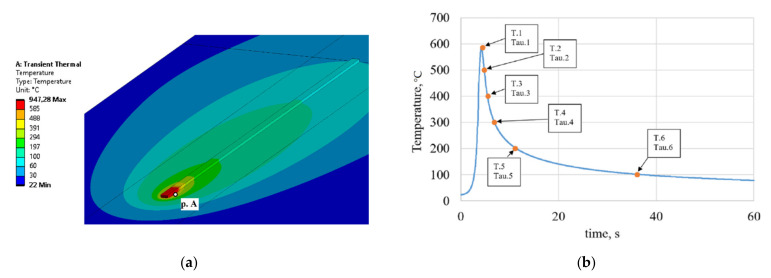
(**a**) Isotherms during deposition (point A–the point on the welding pool boundary); (**b**) thermal cycle of the welding pool boundary for point A.

The obtained agreement between the calculated and experimental data makes it possible to obtain a thermal cycle for the weld pool boundary with acceptable accuracy. Isotherms during deposition simulation are shown in Figure 7a. The thermal cycle for the welding pool boundary (point A in Figure 7a) is shown in Figure 7b.

To estimate the average cooling rate, the ranges of temperature values from the moment of maximum heating (T1 = 585 °C) were selected. Key temperature points are also marked on the thermo cycle for point A.

The crystallization interval at non-equilibrium conditions for alloy 1575 is ~195 °C, and is in the temperature range of 637–445 °C [23]. The cooling rate in this range for the first bead was 225.8 °C/s, which confirms the formation of a supersaturated solid solution [15,16]. Cooling rates for other temperature intervals are presented in Table 4.

**Figure 8:**
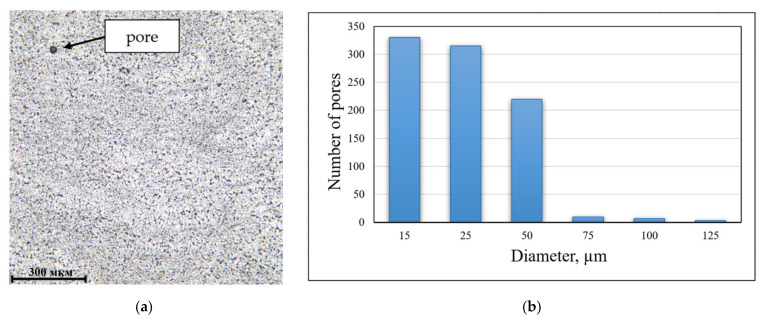
(**a**) Microstructure of specimens aged at 300 °C/6 h (100×); (**b**) cross-sectional pore distribution.

The achievement of the required cooling rate is confirmed by the results of metallography. The microstructure of the specimens is equiaxed fine-grained with moderate porosity, as shown in Figure 8a. The most of pores are from 15 to 50 µm, and individual pores could be up to 125 µm in diameter (Figure 8b). The relative porosity of the specimens is 0.3%. Such porosity should be considered as not affecting the strength as the percentage of weakening of the section from pores is low, and the sizes of individual pores do not lead to a significant increase in stress concentration. Additionally, the lack of influence of such a low porosity on mechanical properties is confirmed by the data obtained in [24]. Therefore, the deposited specimens can be subjected to further mechanical testing.

**Figure 9:**
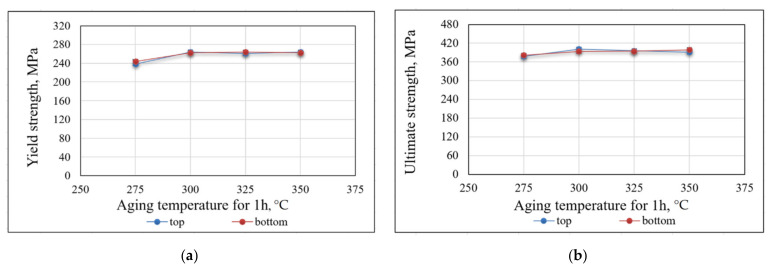
Mechanical properties of specimens, aged at various temperatures for 1 h: (**a**) yield strength; (**b**) ultimate strength.

It was determined that the location of the specimens relative to the substrate (“bottom” or “top”) does not significantly affect the mechanical properties (Figure 9a,b). The spread of the mechanical properties for the same heat treatment is in the range of less than 3%, which can be considered a random error of the experiment. Therefore, specimens with a different location relative to the substrate were considered to be obtained in the same conditions in a further experiment.

The average results for each series of tensile testing are presented in Table 5. Tensile testing of specimens without heat treatment confirms the presence of a quenched structure in the deposited metal. These specimens showed a high level of plasticity (El = 27%) with low level of strength (YS = 176 MPa, UTS = 337 MPa) (Table 5). After heat treatment, specimens showed significantly higher strength properties, with a slight decrease in plasticity. A noticeable effect of mechanical properties increasing was already observed after treatment at 275 °C for 1 h (YS = 241 MPa, UTS = 380 MPa, El = 21%). The highest strength was obtained at 300 °C for 6 h; the yield strength was increased by 52% (to 268 MPa) and the ultimate strength by 20% (to 403 MPa); the level of plasticity remained at the high level (El = 20%).

**Figure 10:**
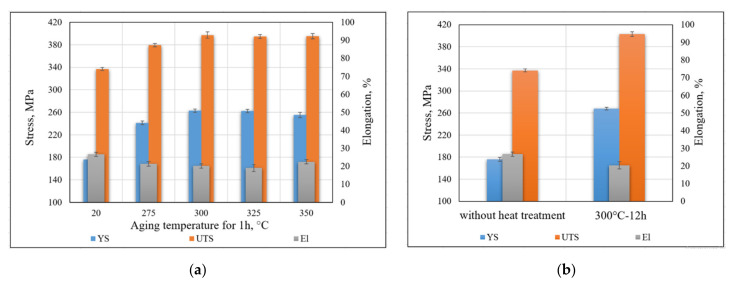
(**a**) Mechanical properties at various temperatures for 1 h aging; (**b**) maximum strength properties due to heat treatment.

A comparison of the specimens tensile testing results with aging for 1 h at various temperatures is shown in Figure 10a. Improvement of the mechanical properties remains noticeable up to 300 °C. Further heating to 325–350 °C does not provide an additional increase in mechanical properties. A comparison of the tensile testing results of specimens with a constant aging temperature for various treatment times is presented in Table 5. The treatment time barely affects the degree of strengthening. The increase in the values of mechanical properties at 3, 6, and 12 h, compared with 1 h, does not exceed 1.9%, which can be described as an experimental error.

Therefore, the optimal heat treatment for WAAM 1575 alloy deposits is aging at 300 °C for 1 h, with the goal of obtaining high strength, good plasticity, and minimum energy costs. Formally, the maximum strength was obtained with aging at 300 °C for 12 h (Figure 10b).

**Figure 11:**
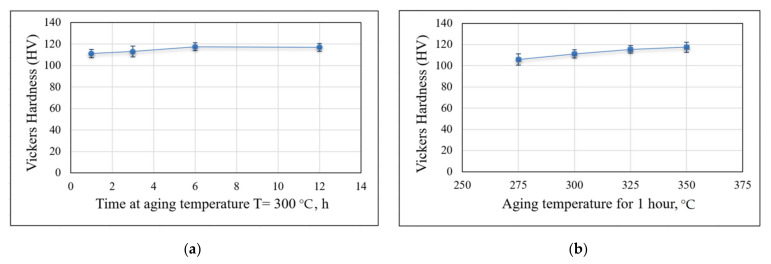
Average values of hardness at various heat treatments: (**a**) at various aging times and a constant temperature of 300 °C; (**b**) at various aging temperatures for 1 hour.

The hardness measurement results of specimens with various heat treatments are presented in Table 6. The hardness values showed an agreement with the results of mechanical tensile tests. After heat treatment, the hardness was increased by more than 19% (from 89 HV to >106). According to the plots in Figure 11a,b, the value of hardness weakly depends on the heat treatment. The plots have practically zero slopes and can be considered almost horizontal.

The coefficient of variation for UTS and YS does not exceed 2.1%; for hardness does not exceed 5.1%, which indicates the consistency of test results and the absence of gross errors in the experiment. The coefficient of variation for elongation does not exceed 23.1%, which indicates an acceptable consistency of the experimental results. The variation in the experimental data of elongation is probably due to the nonuniformity of mechanical properties and the presence of pores, which show themselves as fracture nuclei.

According to mechanical properties, the supersaturated solid solution of Sc in Al was obtained. The structure of beads is quenched and prone to heat treatment, i.e., aging. The data of mechanical properties indicate the beginning of supersaturated solid solution decomposition at 275 °C for 1 h. This result is in good agreement with other studies [15,17], which indicate that the solid solution is stable up to a temperature of 250 °C. Significant growth of mechanical properties continues up to 300 °C for 1 h; a further increase in the temperature and aging time gives a much smaller UTS and YS growth. It can be explained by the almost complete precipitation of the Al3Sc phase from solid solution at a temperature of 300 °C. Formally, the maximum in mechanical properties was achieved at 300 °C for 12 h, at which the yield strength was 268 MPa.

Unfortunately, little research work has been devoted to the WAAM of the Al-Mg-Sc-Zr alloy. However, the addition of scandium and zirconium to Al-Mg alloys is widely used for welding joints. Various welding methods, mainly metal-inert gas welding (MIG) [25], gas-tungsten arc welding (GTA) [12], laser beam welding [26], and even friction stir welding (FSW) [27], were employed in these researches to determinate the mechanical properties for welding joins with these alloys. All these methods confirmed the high mechanical properties of welded joints and good weldability. Successful application of Sc and Zr to Al-Mg fusion welding allows us to use this experience in WAAM.

A similar experimental study was conducted by AML Technologies in Adelaide, Australia [8] to research the effect of various scandium and zirconium additions to WAAM 5XXX alloy deposits. The comparison of mechanical properties with this study is presented in Table 7.

In [8], the yield strength was increased to 259 MPa, which can be considered comparable with the yield stress obtained in this study. However, in [8] this value was achieved using a higher scandium concentration of 0.41% Sc, while the concentration in this study is 0.23% Sc. The alloy 5183, with the addition of 0.26% Sc + 0.1% Zr [8], is the closest to Sv1575 of the content of transition metals Sc and Zr. However, the yield strength of WAAM 5183 alloy deposits was 169 MPa, which is more than 1.5 times lower than deposits from Sv1575.

Potentially, it is possible to increase the mechanical properties of the deposited metal by greater alloying, however the alloying parameters of Sc and Zr should be selected coordinately.

## 4. Conclusions

The research presented in this paper can be summarized, as follows:

1. The supersaturated solid solution, after deposition that is prone to further aging, can be obtained without additional conditions of cooling;
2. The high yield strength of 268 MPa and ultimate strength of 403 MPa of WAAM 1575 alloy deposits were obtained. The welding wire, with a complex addition of scandium and zirconium, expands the application area of WAAM and makes it possible to manufacture large-sized aluminum products with high mechanical properties with all advantages inherent in this technology;
3. The optimal heat treatment is aging at 300 °C for 1 h from the point of view of obtaining high strength properties, good plasticity, and minimum energy costs;
4. Potentially, the mechanical properties can be increased by means of greater alloying and the achievement of a higher cooling rate at deposition.

## Figures and Tables

**Table 1:** Chemical compositions of the welding wire were purchased commercially from LTD Experimental plant «Avial» and Al-6Mg substrate.

Alloy	Mg	Mn	Zr	Sc	Cr	Ti	Ni	Fe	Si	Cu	Al
Sv1575	5.82	0.42	0.19	0.23	0.051	0.028	0.013	0.12	0.05	0.058	Balance
Al-6Mg (substrate)	5.8–6.8	0.5–0.8	-	-	-	0.02–0.1	-	0.4	0.4	0.1	Balance

**Table 2:** Process parameters for each layer.

Layer	Travel Speed, cm/min	Wire Feed Speed, m/min	Current, A	Arc Voltage, V	Pause Time between Layers, s
1	48	8.5	125	14	20
2	60	8.8	127	15	60
3	45	5.5	78	11	180
4	45	5.5	79	12	180
5–15	48	5.5–5.6	78–79	11–12	180

**Table 3:** The heat source parameters.

Parameter	Value
R_x_, mm	6
R_y_, mm	7.5
Q, W	1750
q_max_, W/mm^2^	37.1

**Table 4:** Average cooling rates for temperature ranges.

№	Temperature, °C	Time, s	Average Cooling Rate, (ΔT/ΔTau) °C/s
1	T.1 = 585	Tau.1 = 4.5	283.3
	T.2 = 500	Tau.2 = 4.8	
2	T.1 = 585	Tau.1 = 4.5	168.2
	T.3 = 400	Tau.3 = 5.6	
3	T.1 = 585	Tau.1 = 4.5	118.7
	T.4 = 300	Tau.4 = 6.9	
4	T.1 = 585	Tau.1 = 4.5	57.9
	T.5 = 200	Tau.5 = 11.1	
5	T.1 = 585	Tau.1 = 4.5	15.3
	T.6 = 100	Tau.6 = 36.1	

**Table 5:** Average values of the mechanical properties.

Heat Treatment	Specimens Number	Values	UTS, MPa	YS, MPa	El, %
-	8	A ^1^	337	176	27
		SD ^2^	2.8	3.7	1.2
		CV ^3^	0.8%	2.1%	4.4%
275 °C-1 h	4	A	380	241	21
		SD	2.8	3.3	1.3
		CV	0.7%	1.4%	6.2%
275 °C-3 h	4	A	381	253	16
		SD	8.1	1.9	3.7
		CV	2.1%	0.8%	23.1%
275 °C-6 h	2	A	388	259	18
		SD	3.3	3.7	2.4
		CV	0.9%	1.4%	13.3%
300° C-1 h	4	A	397	263	20
		SD	5.5	3.1	1.2
		CV	1.4%	1.2%	6.0%
300 °C-3 h	4	A	398	265	20
		SD	1.5	2.5	1.2
		CV	0.4%	0.9%	6.0%
300 °C-6 h	3	A	401	268	20
		SD	5.3	4.4	4.3
		CV	1.3%	1.6%	21.5%
300 °C-12 h	3	A	403	268	20
		SD	4.0	2.4	2.1
		CV	1.0%	0.9%	10.5%
325 °C-1 h	4	A	395	262	19
		SD	3.4	3.0	2.0
		CV	0.9%	1.1%	10.5%
325 °C-6 h	2	A	402	261	22
		SD	1.3	2.1	4.0
		CV	0.3%	0.8%	18.2%
350 °C-1 h	4	A	395	255	24
		SD	4.7	4.6	1.2
		CV	1.2%	1.8%	5.0%

^1^ A—Average, ^2^ SD—Standard deviation, ^3^ CV—Coefficient of Variation.

**Table 6:** Average values of hardness.

Aging Temperature, °C	Aging Time, h	Hardness, HV	Standard Deviation	Coefficient of Variation, %
-	0	89	2.1	2.4
275	1	106	5.4	5.1
300	1	111	3.8	3.4
	3	113	5.1	4.5
	6	118	3.7	3.1
	12	117	3.7	3.2
325	1	115	3.6	3.1
350	1	118	4.8	4.1

**Table 7:** Tensile testing results of WAAM deposits at various Sc and Zr additions to Al-Mg system welding wires.

Alloy		Without Heat Treatment			Heat Treatment	With Heat Treatment			Reference
		UTS, MPa	YS, MPa	El, %		UTS, MPa	YS, MPa	El, %	
5183	+0.24 Sc	321	161	25	300 °C-1 h	335	214	19	[8]
	+0.41 Sc	350	212	22	300 °C-5 h	377	259	17	
	+0.075 Sc	268	119	22	no data	271	125	18	
	+0.11 Zr								
	+0.26 Sc	306	161	18		312	169	22	
	+0.1 Zr								
5356	+0.23 Sc	323	179	21		337	196	24	
Sv1575	+0.23 Sc +0.19 Zr	337	176	27	300 °C-1 h	397	263	20	
					300 °C-6 h	401	268	20	this study
					300 °C-12 h	403	268	20	

## Data Availability

The data presented in this study are available on request from the corresponding author. The data are not publicly available due to commercial intellectual property.
